# Quantitative ctDNA Detection in Hepatoblastoma: Implications for Precision Medicine

**DOI:** 10.3390/cancers16010012

**Published:** 2023-12-19

**Authors:** Smadar Kahana-Edwin, James Torpy, Lucy E. Cain, Anna Mullins, Geoffrey McCowage, Sarah E. Woodfield, Sanjeev A. Vasudevan, Dan P. T. Shea, Andre E. Minoche, Andres F. Espinoza, Sarah Kummerfeld, Leonard D. Goldstein, Jonathan Karpelowsky

**Affiliations:** 1 Children’s Cancer Research Unit, Kids Research, The Children’s Hospital at Westmead, Sydney, NSW 2145, Australia; 2 Kinghorn Centre for Clinical Genomics, Garvan Institute of Medical Research, Sydney, NSW 2010, Australia; 3 Cancer Centre for Children, The Children’s Hospital at Westmead, Sydney, NSW 2145, Australia; 4 Divisions of Pediatric Surgery and Surgical Research, Michael E. DeBakey Department of Surgery, Pediatric Surgical Oncology Laboratory, Texas Children’s Surgical Oncology Program, Texas Children’s Liver Tumor Program, Dan L. Duncan Cancer Center, Baylor College of Medicine, Houston, TX 77030, USA; 5 St Vincent’s Clinical School, UNSW Sydney, Sydney, NSW 2217, Australia; 6 Paediatric Oncology and Thoracic Surgery, The Children’s Hospital at Westmead, Sydney, NSW 2145, Australia; 7 Division of Child and Adolescent Health, The University of Sydney, Sydney, NSW 2050, Australia

**Keywords:** hepatoblastoma, ctDNA, NGS, AFP, ddPCR, liquid biopsy

## Abstract

**Simple Summary:**

Driver mutations in *CTNNB1* are a hallmark of hepatoblastoma and offer a common biomarker for a liquid biopsy approach that is based on the presence of *CTNNB1* circulating tumor DNA (ctDNA). We provide promising evidence for the utility of quantitative *CTNNB1* ctDNA detection in hepatoblastoma for dynamic tumor burden and treatment response monitoring, compared with the current clinical indicators and biomarkers for this disease.

**Abstract:**

Hepatoblastoma is characterized by driver mutations in *CTNNB1*, making it an attractive biomarker for a liquid biopsy approach utilizing circulating tumor DNA (ctDNA). This prospective observational study sought to ascertain the feasibility of ctDNA detection in patients with hepatoblastoma and explore its associations with established clinical indicators and biomarkers, including serum Alpha-fetoprotein (AFP). We obtained 38 plasma samples and 17 tumor samples from 20 patients with hepatoblastoma. These samples were collected at various stages: 10 at initial diagnosis, 17 during neoadjuvant chemotherapy, 6 post-operatively, and 5 at disease recurrence. Utilizing a bespoke sequencing assay we developed called QUENCH, we identified single nucleotide variants and deletions in *CTNNB1* ctDNA. Our study demonstrated the capability to quantitate ctDNA down to a variant allele frequency of 0.3%, achieving a sensitivity of 90% for patients at initial diagnosis, and a specificity of 100% at the patient level. Notably, ctDNA positivity correlated with tumor burden, and ctDNA levels exhibited associations with macroscopic residual disease and treatment response. Our findings provide evidence for the utility of quantitative ctDNA detection in hepatoblastoma management. Given the distinct detection targets, ctDNA and AFP-based stratification and monitoring approaches could synergize to enhance clinical decision-making. Further research is needed to elucidate the interplay between ctDNA and AFP and determine the optimal clinical applications for both methods in risk stratification and residual disease detection.

## 1. Introduction

Hepatoblastoma is the most common liver tumor diagnosed in children, predominantly in young children under the age of 3 years. Hepatoblastoma is a rare cancer type with an approximate annual incidence of two cases per million persons, and it has a rising incidence of about 2.5% per year. Its management typically involves pre- and/or post-operative chemotherapy, tumor resection with partial hepatectomy or, in selected cases, liver transplantation [1]. Risk assessments and subsequent therapy planning are essential for achieving favorable outcomes. To address this, international risk classification standards were created based on the PRE-Treatment EXTent of tumor (PRETEXT) imaging staging system [2], serum Alpha-fetoprotein (AFP) values, and patient age to separate patients into very low-risk, low-risk, moderate-risk, and high-risk groups [3]. Employing a risk-adapted chemotherapy strategy alongside advanced surgical approaches such as indocyanine green (ICG) fluorescence-guided surgery and liver transplantation has significantly improved survival rates in children with hepatoblastoma, especially in the high-risk group [4]. Despite these advancements in treatment strategies, approximately 12% of patients in complete remission are susceptible to liver and/or lung relapses [1], underscoring the need for improved measurable residual disease (MRD) detection and treatment options. Furthermore, some of the most high-risk tumors, especially relapse tumors, do not secrete AFP and thus lack the AFP biomarker [5]. By contrast, this risk stratification system lacks efficacy to distinguish between the prognosis of the very low-risk and low-risk groups [6] and could benefit from further development. This is needed, as the distinction between low-risk and very low-risk groups determines the use of adjuvant chemotherapy post-operatively in the former or observation only for the latter, which is critical in the context of morbidity associated with platinum-based chemotherapy in young children.

Cell-free circulating tumor DNA (ctDNA), also known as ‘cancer liquid biopsy’, serves as a surrogate for the tumor genome and is often the most accessible and least invasive clinical sample for applications such as therapy selection, post-treatment monitoring, and early cancer screening [7,8]. Ensuring a high assay sensitivity and precise quantitative assessment of ctDNA is imperative for delivering an accurate assessment of disease burden. While hepatoblastoma typically has a low mutational burden, a frequent recurrent alteration is the mutation or deletion of exon 3 of the *CTNNB1* gene [9,10,11,12]. We previously reported an association between *CTNNB1* ctDNA digital droplet PCR (ddPCR) variant allele frequency (VAF) and serum AFP levels during treatment in a small series of three cases [13]. All three patients were diagnosed with localized hepatoblastoma and tested positive for ctDNA at multiple time points (ranging from 2 to 6) before undergoing surgical resection of their primary tumors. Longitudinal sampling during treatment, evaluating the dynamics of *CTNNB1* mutations, enabled the monitoring of disease status with minimal invasiveness. However, utilizing patient-specific ddPCR assays poses a challenge for real-time data at diagnosis due to varying mutation locations and length information that can only be determined by the sequencing of tissue biopsies.

To address these challenges, we developed a **q**uantitative **u**niv**e**rsal **n**ext-generation sequencing (NGS) **c**tDNA assay for **h**epatoblastoma (QUENCH), enabling near real-time tumor-agnostic ctDNA evaluation. In a cohort of 38 samples from 20 patients, QUENCH’s effectiveness in detecting *CTNNB1* ctDNA was assessed and compared with ddPCR ctDNA and AFP levels, evaluating correlations with tumor burden and treatment response.

## 2. Materials and Methods

### 2.1. Study Design

The study was conducted under research protocols approved by the Sydney Children’s Hospitals Network and Baylor College of Medicine Human Research Ethics Committees (reference numbers HREC/17/SCHN/302 and BCM IRB H-38834, respectively), with informed consent obtained from all participants.

Eligibility criteria included a diagnosis of hepatoblastoma and available plasma samples collected at diagnosis or during treatment and surveillance. Accordingly, 20 patients were enrolled and 38 samples collected prospectively across the two sites: 25 samples from 9 patients treated at The Children’s Hospital at Westmead, and 13 samples from 11 patients treated at Texas Children’s Hospital.

An additional thirteen samples were collected at The Children’s Hospital at Westmead from children in long-term remission from solid tumors (*n* = 5 neuroblastoma, *n* = 5 sarcomas, and *n* = 3 hepatoblastoma).

AFP serum levels were routinely tested and obtained from the electronic health record. Additional clinical and pathological information can be found in Supplementary Materials: Table S1.

- Samples collected at The Children’s Hospital at Westmead: 2–3 mL blood samples were collected in 10 mL Streck tubes (Cell-Free DNA BCT^®^, STRECK, La Vista, NE, USA, catalog No. 218997) and processed for plasma at ambient temperature in a double-centrifugation protocol as previously described [13]. Plasma aliquots were stored at −80 °C until DNA isolation.
- Samples collected at Texas Children’s Hospital: 2–3 mL blood samples were collected in EDTA tubes and processed for plasma in a double-centrifugation protocol: a first centrifugation at 1200× *g* for 10 min followed by plasma supernatant aspiration into new tubes without disturbing the buffy coat layer, then a second centrifugation of the plasma supernatant at 15,000 rpm at 4 °C for 10 min, followed by aspirating the top phase into new tubes without disturbing the pellet, and storing at −80 °C until DNA isolation.

### 2.2. DNA Isolation and Quantification

- Cell-free DNA (cfDNA) was extracted from 0.5 to 1 mL of frozen plasma samples using the QIAamp Circulating Nucleic Acid kit (Qiagen, catalog No. 55114, Chadstone, VIC, Australia) according to the manufacturer’s instructions, except for increasing the proteinase digest step to 60 min for plasma samples collected in Streck tubes, as recommended by the Streck product literature. DNA was eluted in the 40 µL buffer provided with the kit and stored at −80 °C until analysis. DNA quantification was performed using the Qubit dsDNA High Sensitivity Assay Kit for the Qubit 2.0 Fluorometer (Life Technologies, Carlsbad, CA, USA).
- Genomic DNA was extracted from cell lines using AllPrep DNA/RNA/Protein kit (Qiagen; catalog No. 80004), and normal peripheral mononuclear cells and matched whole blood (germline) samples using QIAGEN DNeasy Blood and Tissue kit (Qiagen; catalog No. 69504), according to the manufacturer’s instructions.

### 2.3. QUENCH

- Library preparation: CfDNA libraries were constructed with a customized QIAseq Targeted DNA Panel Kit (QIAGEN), as described in detail previously [14]. The input amount preferred for library preparation was 40 ng, but 3.5–40 ng cfDNA samples were included in this cohort (mean 19.4 ng). Briefly, cfDNA was end-repaired, A-tailed, and ligated with UMI barcoded adaptors. The adaptor-ligated libraries were target-enriched with PCR using a panel of loci-specific primers (8 cycles). The targeted enrichment was performed with a customized QIAseq Targeted DNA Panel primer design to amplify the region covering exons 2–4 of the *CTNNB1* gene. A double-stranded higher tiling density design was used to accommodate the small cfDNA fragments. The target-enriched libraries were further amplified for 23 cycles with PCR and were size selected for an average fragment size of 300 base pairs (bp) (corresponding to an insert size of about 110 bp). The library profile was quantified using Qubit dsDNA HS Assay kit (Invitrogen™, Thermo Fisher Scientific, Waltham, MA, USA). The quality and quantity of the prepared library was assessed by the Australian Genome Research Facility (AGRF), Sydney, Australia. The library profile was analyzed with the High Sensitivity D1000 ScreenTape System (Agilent Technologies, Palo Alto, CA, USA) and qPCR with the NEBNext Library Quantification Kit (New England Biolabs Pty Ltd., Ipswich, MA, USA). After quantification, the libraries were normalized and pooled in equimolar quantities. Sequencing was performed with the Illumina MiSeq according to manufacturer’s recommendations using paired-end sequencing (2  ×  150 bp) with the MiSeq v.2 reagent kit and a custom primer (Custom Read primer 1) provided with the QIAseq library kit. The median average read coverage was 685 (range 407–3987) and the median average UMI coverage was 378 (range 98–1942).
- Data analysis: Raw sequence data in FASTQ format were processed with the QIAseq DNA pipeline available at https://github.com/qiaseq/qiaseq-dna, accessed on 19 January 2022. Briefly, after trimming adapter sequences, reads were mapped to the human reference genome hg19 with BWA MEM, and single nucleotide variants (SNVs)/indels were called with smCounter2 [15] with default parameters. Read alignments were used for structural variant (SV) analysis using a custom pipeline. Reads were marked as duplicates based on their genomic position and UMI sequence using Picard MarkDuplicates (v2.26.3). Deletions in the *CTNNB1* gene were identified using a custom R software package SVseek. Briefly, candidate deletions were identified as regions between split read segments, where the region start and end defined two breakpoints. In the case of ambiguous nucleotides mapping to either segment, they were assigned to the first segment. The deletion with the largest number of supporting reads was retained. Deletion VAFs were estimated based on informative reads at each breakpoint (Supplementary Materials Figure S1). For breakpoint *i*:
  (1) $${VAF}_{i}=\frac{s_{i}}{t_{i}}$$
  where the total number of reads at breakpoint *i* was *t_i_ = s_i_ + n_i_*. *s_i_* and *n_i_* indicate the number of deletion-supporting and non-supporting read pairs, respectively. Reads used for VAF calculation at breakpoint *i* were required to have read 1, containing the gene-specific primer, overlap the non-deleted region flanking breakpoint *i*. Reads were considered deletion-supporting or non-supporting depending on whether read 2 overlapped the non-deleted region flanking the second breakpoint, or the deleted region, respectively. Read overlap was required to be at least 19 bp, the seed length used for BWA-MEM alignment. A final VAF estimate was obtained as the weighted average of the breakpoint estimates:
  (2) $$VAF=w_{1}{VAF}_{1}+w_{2}{VAF}_{2}$$
  where *w_i_* = $\frac{ti}{t1+t2}$.

### 2.4. QUENCH Limit of Detection (LoD)

DNA from the hepatoblastoma cell line HepG2, harboring the 548 bp deletion CTNNB1:c.73_420del [16], was mixed with a wild type *CTNNB1* genome to produce artificial VAF levels of 0%, 0.03%, 0.1%, 0.2%, 0.3%, 1%, 1.2%, 5%, 25%, and 50%. We used a clinically relevant DNA input amount of 20 ng (see Section 3.7) and sequenced to a median average read depth of 627x (407–837x) and median average UMI depth of 307x (221–430x) across *CTNNB1*. A deeper sequencing of libraries with shallow read depth did not result in new detections, suggesting sensitivity was limited by library complexity.

The LoD was determined as the lowest VAF that could be detected with a minimum of 1 variant copy for SV calling. Given that 20 ng of DNA represents about 3030 diploid human genome equivalents [17], the theoretical maximum expected sensitivity was 0.03% VAF. Given the observed maximum average coverage of 837x, a more realistic detection threshold is a VAF of about 0.12. Accordingly, we observed a linear detection of the variant down to a VAF of 0.1% (R^2^ = 0.9953), and we were not able to detect the variant at 0.03% or beyond that level, setting the LoD at 0.1% VAF (Supplementary Materials Figure S2).

### 2.5. QUENCH Limit of Blank

Here, the limit of blank (LoB) represents the highest number of reads erroneously supporting a *CTNNB1* deletion in samples containing mutation-negative *CTNNB1* [18]. To provide the QUENCH LoB, a 20 ng input DNA from 4 samples with a wild type *CTNNB1* were used: the cfDNA sample from a patient with hepatoblastoma (Supplementary Materials: Table S1), and the three cell lines A673, ES8, and Kelly.

### 2.6. ddPCR

Mutant (MT) and wild type (WT) *CTNNB1* sequences were used for designing ddPCR (Bio-Rad Laboratories, Hercules, CA, USA) assays following the dMIQE guidelines [19] (Supplementary Materials: Table S2). ddPCR reactions were assembled using the standard protocol as previously described [13]: the ddPCR reaction consisted of 10 μL ddPCR™ Supermix for Probes (No dUTP) (Bio-Rad Laboratories), 900 nM/reaction of primers mix for both MT and WT *CTNNB1*, 250 nM/reaction of probes mix for both MT and WT *CTNNB1*, and 4 units of restriction enzyme (HaeIII or MseI, New England BioLabs, as per Supplementary Materials: Table S2), for a final volume of 20 μL. CfDNA was added in the same amount as was used for the QUENCH library preparation. Non-template controls (NTCs) contained purified water instead of cfDNA. Tm was optimized for each of the assays using gradient PCR on matched tumor DNA. The assays’ specificity was determined on matched whole blood (germline) genomic DNA as well as genomic DNA from 2 to 5 different pediatric cell lines.

The ddPCR reaction mixture was used for droplet generation, and ddPCR was performed using the QX200 ddPCR system according to the manufacturer’s instructions (Bio-Rad Laboratories). QuantaSoft™ Analysis Pro v1.0 software (Bio-Rad Laboratories) was used for data analysis. Each sample was tested in 2 to 3 replicates performed in at least 2 different experiments.

HepG2 CTNNB1.p.W25_I140del mutation status was confirmed by Sanger sequencing. The relative copy number of both MT and WT alleles was determined by comparing to a normal genome from a healthy individual.

### 2.7. Statistical Analysis

Linear regression was performed using Excel Microsoft Office Professional Plus 2013. A Mann–Whitney nonparametric t test and ordinary one-way ANOVA were performed using GraphPad Prism version 9.3.1 for macOS (GraphPad Software, San Diego, CA, USA, www.graphpad.com, accessed on 11 December 2023). Sensitivity was calculated as the fraction of positive samples that were classified as positive by the alternative method. Specificity was calculated as the fraction of negative samples that were classified as negative by the alternative method.

## 3. Results

### 3.1. Characteristics of the Study Population

Twenty patients diagnosed with hepatoblastoma were included in this study. The median age at diagnosis was 24 months (ranging from 3 months to 14 years old), 8/20 (40%) were female, and 6/20 (305) had metastatic disease at diagnosis. In 11 patients with available histological classification, 9 (82%) were classified as having mixed epithelial and mesenchymal histology. In 10 patients with available AFP levels at the time of diagnosis, the mean value was 140,223 kIU/L (compared to age-adjusted normal values of AFP ranging from 1 to 175 kIU/L [20,21,22]).

Somatic mutations in the exon 3 region of the *CTNNB1* gene were evaluated with Sanger sequencing of the primary tumors or metastases, as previously described [13]. Data were available for 18 of the 20 patients, revealing the presence of seven missense SNVs, ten deletions ranging from 10 to 348 bp, and one instance of a wild-type *CTNNB1* (Supplementary Materials Figure S3).

### 3.2. CtDNA Positivity and VAF Levels Correlate with Macroscopic Residual Disease

The scarcity of ctDNA and the extremely low amount of cfDNA (in the ng range) might hinder the successful sampling of rare variants. To develop and benchmark our assay to detect low fraction somatic variants in plasma, the sensitivity, specificity, and linearity were determined at low variant levels, and an LoD of 0.1% VAF and LoB of 0% VAF were calculated (see Section 2). No false positives were observed.

**Figure 1:**
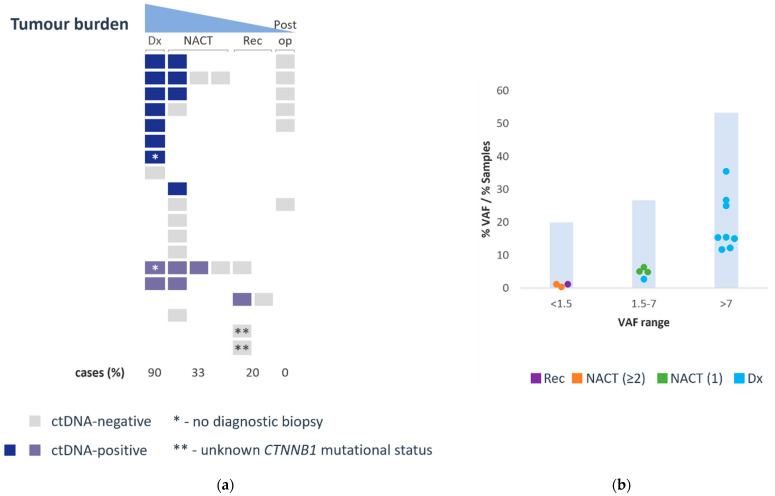
CtDNA positivity correlates with tumor burden (**a**) and levels correlate with clinical time point (**b**). (**a**) Each row represents a different patient. Colored rectangles represent tested cfDNA samples: grey—ctDNA-negative, indigo—ctDNA-positive in localized cases, purple—ctDNA-positive in metastatic cases. (**b**) Scatter plot of ctDNA-positive samples at different VAF ranges, color coded according to time point of collection. Dx—initial diagnosis—cyan; NACT—during neoadjuvant chemotherapy (the number of previously administered cycles shows in brackets)—green (post 1 cycle)/orange (post 2 or more cycles); Rec—disease recurrence—purple. CtDNA VAF (for the scatter plot) and % of samples in each VAF range presented in bar chart.

QUENCH demonstrated its ability to identify *CTNNB1* variants from ctDNA in 65% (11/17) of cases, with Sanger sequencing confirming *CTNNB1* mutations, encompassing eight SV and three SNVs. The VAF ranged from 0.3% to 35.4%, with 20% of samples showing VAF below 1.5%. Importantly, the NGS-based *CTNNB1* variant calling was conducted independently of Sanger sequencing, and where variants were identified by both methods, they were entirely concordant, validating the assay’s accuracy. Notably, *CTNNB1* variants were detectable in 90% (9/10) of samples collected at the initial diagnosis stage, exhibiting a mean VAF of 17.7% (ranging from 2.7% to 35.4%). During or after neoadjuvant chemotherapy, 33–36% (5/15 to 5/14, as one case was lacking tumor *CTNNB1*-mutation confirmation with Sanger sequencing) of samples had detectable *CTNNB1* variants, with a mean VAF of 3.5% (ranging from 0.3% to 6.3%). In cases of metastatic recurrence, *CTNNB1* variants were identified in 20–33% (1/5 to 1/3, as only 3 of the 5 cases had a Sanger-sequencing-confirmed tumor *CTNNB1*-mutation) of samples, with a VAF of 1.1%. However, no *CTNNB1* variants were observed post-operatively in fully resected localized disease (0/6). These findings suggest a positive correlation between ctDNA positivity and tumor burden (Figure 1a), as well as a correlation between VAF levels and tumor burden (Figure 1b).

### 3.3. Assay Verification—Concordance with ddPCR

**Figure 2:**
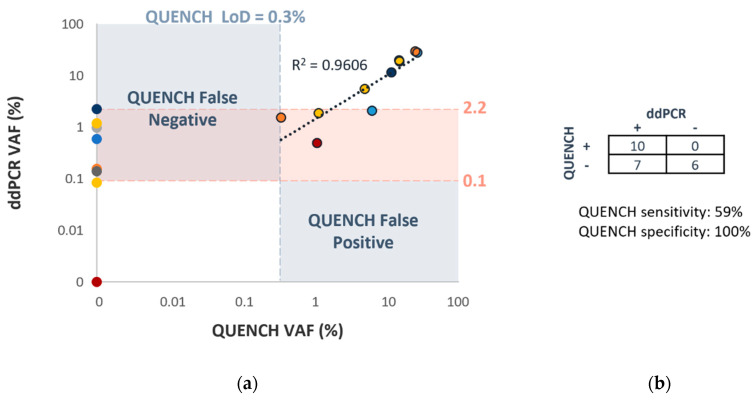
QUENCH verification—concordance with ddPCR. Matched ddPCR and NGS results from 8 patients (color-coded according to each patient). (**a**) High concordance (R^2^ = 0.9606) observed in samples with QUENCH VAF > 0.3%. ddPCR shows higher sensitivity for VAF < 2.2%. (**b**) Sensitivity, specificity, and concordance of variant detection evaluated by QUENCH and ddPCR.

To validate the quantitation capability and evaluate the sensitivity of QUENCH in clinical samples, we conducted a comparative analysis with ddPCR. A total of 23 samples from 8 patients, evenly divided between those with SNVs and deletions in their primary tumors (Figure 2), were included in the analysis. In cases where VAF exceeded 0.35%, QUENCH and ddPCR exhibited excellent concordance, with a strong correlation (R^2^ = 0.9606, Figure 2a). However, QUENCH’s sensitivity appeared inconsistent within the VAF range of 0.1% to 2.2%. Although the theoretical LoD for QUENCH was established at 0.1%, only four samples were detected by QUENCH in this range, while ddPCR identified six additional samples that QUENCH missed. Notably, there were no instances where QUENCH yielded positive results while ddPCR showed negative results, and six samples yielded negative results with both QUENCH and ddPCR. In comparison to ddPCR, QUENCH demonstrated a sensitivity of 59% and a specificity of 100% (Figure 2b).

### 3.4. CtDNA Correlation to AFP

AFP levels were abnormal in 34 out of 36 samples with available information. Generally, the ctDNA levels measured using QUENCH did not exhibit a strong correlation with AFP levels, as 17 samples were found to be ctDNA-negative despite elevated AFP levels. Notably, four of these samples were collected after complete resection with clear margins of localized tumors. These samples were expected to be negative for hepatoblastoma markers, though it is possible that microscopic residual disease was present and eliminated by subsequent adjuvant chemotherapy. It is important to mention that AFP levels in these cases eventually returned to the reference range, but only after 2 to 9 months post-operation, owing to AFP’s half-life of 4–9 days [21,22,23].

**Figure 3:**
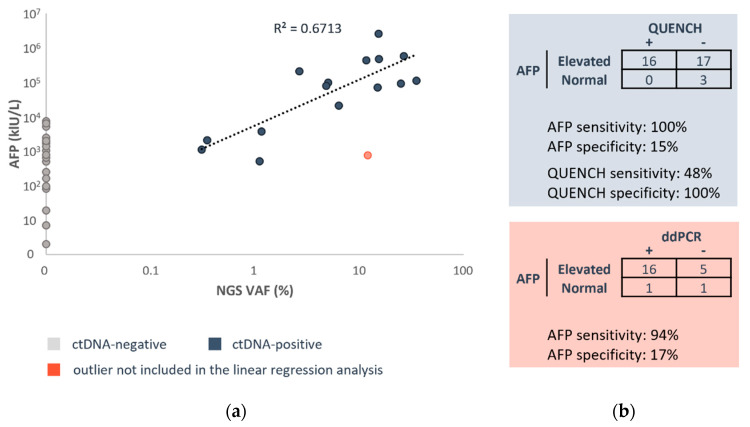
CtDNA moderately correlates with AFP. (**a**) VAF of QUENCH-positive samples and AFP are moderately concordant (R^2^ = 0.6713). Grey—ctDNA-negative, indigo—ctDNA-positive, salmon—ctDNA-positive outlier not included in the linear regression analysis. (**b**) Sensitivity, specificity, and concordance of variant detection evaluated by QUENCH and AFP and ddPCR and AFP.

To explore this further, we examined the relationship between 15 QUENCH-positive samples and AFP levels. We found that ctDNA VAF correlated with the order of magnitude of AFP levels in most cases (R^2^ = 0.6713, Figure 3a). However, there was one exception, where the sample was collected from a patient diagnosed with hepatoblastoma at an unusually older age (14 years). Although AFP was very sensitive, in terms of diagnostic specificity, AFP exhibited lower values compared to both QUENCH and ddPCR (15% and 17%, respectively: Figure 3b).

### 3.5. AFP and ctDNA Correlate with Tumor Size

**Figure 4:**
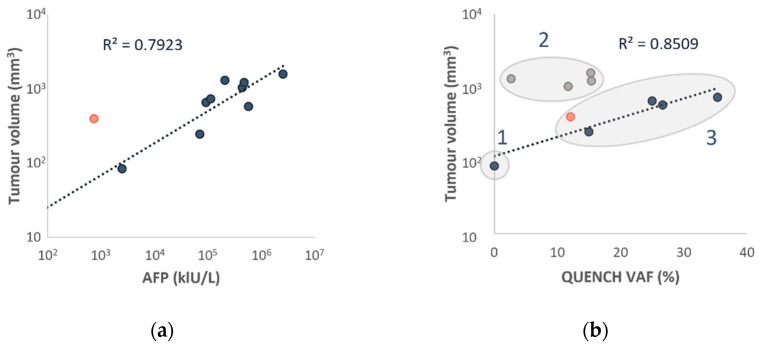
AFP levels correlate with tumor size at diagnosis (**a**), but only smaller tumors show a correlation between ctDNA VAF and tumor size (**b**). Matched tumor volume, AFP, and NGS results at diagnosis from 10 patients. (**a**) AFP levels and tumor size are concordant (R^2^ = 0.7923). Indigo—samples included in the analysis, salmon—sample not included in the analysis. (**b**) CtDNA VAF levels and small tumor size are concordant (R^2^ = 0.8509). Indigo and salmon—samples included in the analysis, grey—samples excluded from the linear regression analysis.

Diagnostic MRI or CT imaging studies and cfDNA samples were available for 10 patients, with an average total tumor volume of 780 cm^3^ (ranging from 83 cm^3^ to 1574 cm^3^). A strong linear correlation was found between AFP levels and the volumetric assessment of tumor size, apart from one patient—an older patient, consistent with the previously mentioned outlier (R^2^ = 0.7923, Figure 4a).

A different pattern was observed for ctDNA levels. Patients were categorized into three groups based on tumor size and ctDNA VAF. Group I comprised a patient with a small tumor (83 cm^3^) and undetectable ctDNA. Group II consisted of patients with moderate ctDNA VAF and significantly larger tumors (ranging from 2.7% to 15.4% VAF and 1031 cm^3^ to 1574 cm^3^ in tumor size). Group III included patients with high ctDNA VAF and medium-sized tumors (ranging from 12.1% to 35.4% VAF and 245 cm^3^ to 731 cm^3^ in tumor size).

Upon excluding patients from Group II, we identified a robust linear correlation between ctDNA VAF and tumor volume (R^2^ = 0.8509, Figure 4b). However, it is important to note that this correlation did not hold for Group II. Despite our efforts, we could not identify any associations with other clinical, pathological, or radiological parameters (Supplementary Materials Table S1) that could explain the differing propensity to release ctDNA in this group.

### 3.6. CtDNA VAF Correlate with Dynamic Treatment Response

**Figure 5:**
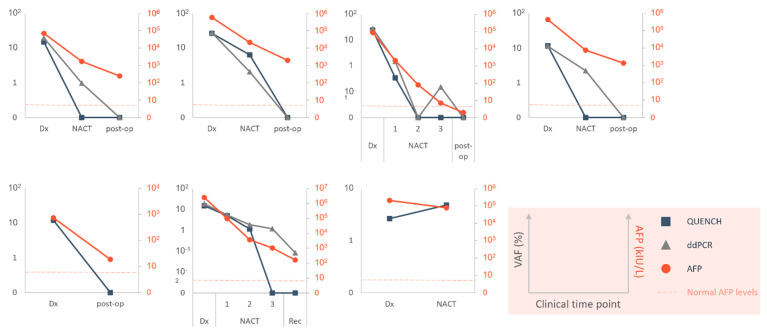
CtDNA correlates with dynamic treatment response. Each graph represents an individual patient. Serial samples with matched ctDNA VAF (evaluated by QUENCH—indigo squares or ddPCR—grey triangles, left Y axis) and AFP levels (salmon, right Y axis), collected at different clinical time points: Dx—initial diagnosis, NACT—during neoadjuvant chemotherapy, post-op—post resection of primary tumor, Rec—disease recurrence. Age-adjusted normal values of AFP presented with a dashed line.

Serial cfDNA samples (n = 23) were collected from seven patients, and the analysis revealed consistent dynamics in both ctDNA and AFP levels in six of these patients, reflecting their response to systemic treatment (Figure 5). The only exception was a sample collected at diagnosis which had a VAF of only 2.7% (compared with eight samples collected at diagnosis with VAF 11.7–35.4%). The sequential sample collected at the time of tumor resection showed a VAF of 4.8%, while serum AFP levels decreased by approximately threefold between these time points. It is worth noting that this sample was collected in an EDTA tube, and we speculate that this discrepancy may be related to the pre-analytical conditions of blood collection and/or processing.

Of note, as ctDNA VAF during treatment was low, ddPCR proved to be a more precise tool for monitoring ctDNA changes, as it successfully identified eight out of nine samples with VAF under 2.2%, whereas QUENCH failed to detect five of them.

### 3.7. CtDNA Correlation to CfDNA

**Figure 6:**
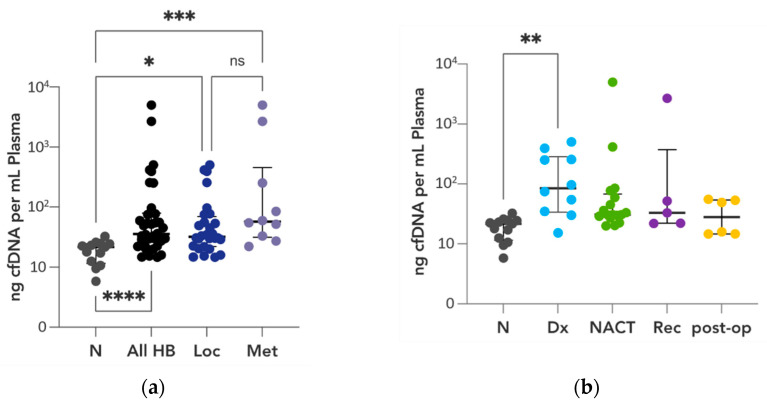
CfDNA levels in hepatoblastoma correlate with disease stage (**a**) and clinical time point (**b**). N—control group, HB—hepatoblastoma, Loc—localized disease, Met—metastatic disease, Dx—initial diagnosis, NACT—during neoadjuvant chemotherapy, Rec—disease recurrent disease, post-op—post resection of primary tumor. *p*-values are presented in asterisks (* = 0.0425, ** = 0.0098, *** = 0.0005, **** < 0.0001, ns—not significant).

In comparison to the control group, patients with hepatoblastoma exhibited significantly elevated levels of cfDNA (45–4992 ng/mL of plasma, median 36 ng/mL; Mann–Whitney test, *p* < 0.0001). Furthermore, cfDNA levels showed a dependence on disease stage, with patients having localized disease presenting a median cfDNA concentration of 32 ng/mL, whereas those with metastatic disease had a median of 57 ng/mL (ordinary one-way ANOVA *p* = 0.0425 and *p* = 0.0005, respectively) (Figure 6a). The time point at which cfDNA samples were collected also had a notable impact. Samples obtained at the time of diagnosis exhibited a median cfDNA concentration of 86 ng/mL, significantly higher than samples collected during neoadjuvant chemotherapy, post operation, or at disease recurrence (ordinary one-way ANOVA, *p* = 0.0098) (Figure 6b).

**Figure 7:**
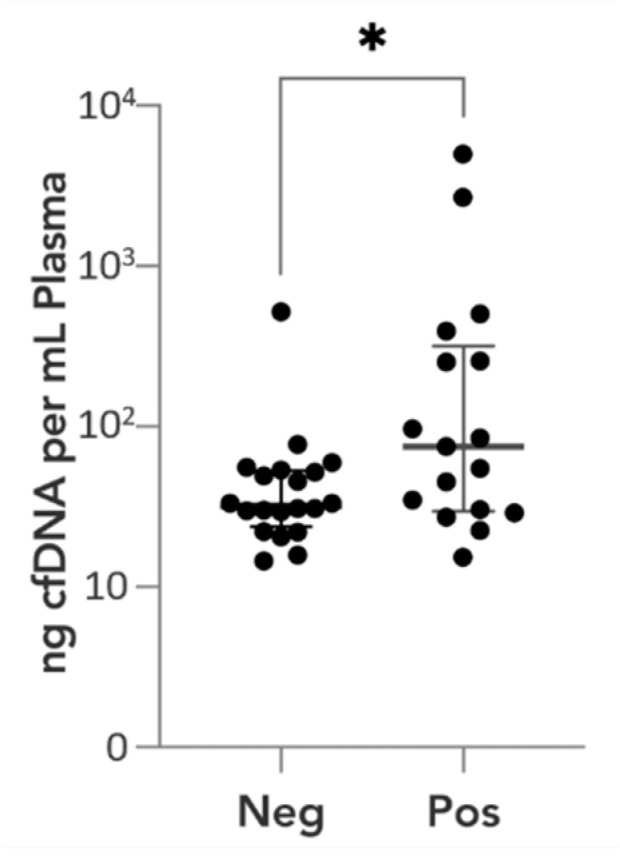
Correlation between cfDNA amount in plasma and ctDNA positivity. CfDNA levels (ng/mL) and cfDNA samples’ positivity (Pos) or negativity (Neg) for *CTNNB1* mutation. *p*-value is presented in asterisks (* = 0.0441).

Mutation-positive cfDNA samples exhibited higher levels of cfDNA than mutation-negative cfDNA samples (15–4992 ng/mL of plasma, median 564 ng/mL vs. 15–520 ng/mL of plasma, median 61 ng/mL; Mann–Whitney test, *p* = 0.0441) (Figure 7, Supplementary Materials: Table S1). Despite the statistical significance, it is noteworthy that a substantial overlap exists between these two groups. Therefore, the observed significance does not translate into predicting ctDNA positivity solely based on cfDNA levels lower than 100 ng/mL.

## 4. Discussion

Liquid biopsies are rapidly gaining traction for their potential to revolutionize cancer diagnosis, disease monitoring, and treatment selection through the blood-based analysis of shed biomolecules [8]. As hepatoblastoma arises almost exclusively in young children, with 90% of cases occurring in children less than five years old and primarily before the age of three [24], a minimally invasive diagnostic tool as liquid biopsy is an attractive prospect [25]. However, at present, very little data comparing ctDNA to AFP in hepatoblastoma are available [13].

There are several potential advantages of ctDNA over AFP testing in hepatoblastoma. The foremost is that ctDNA is tumor-specific, enhancing its reliability as a marker of disease status or to identify the nature of the tumor, in contrast to AFP, which is tumor-associated. Furthermore, being a driver event, all tumor cells are expected to harbor a *CTNNB1* variant, regardless of tumor heterogeneity. Secondly, serum AFP concentrations are highly elevated in neonates and exceedingly high levels can be observed in premature infants due to their developmental stage [26], making AFP an inaccurate measurement in this population. The incidence rate for hepatoblastoma has been increasing in developed countries worldwide in recent decades, by about 2.5% per year [27], and is attributed in part to the increased survival rates of premature babies, as extremely premature babies with a birth weight of less than 1 kg have been reported to have a greatly increased risk of developing hepatoblastoma [28]. Taken together, these could impose a difficulty in interpreting AFP values in a bigger proportion of cases in the future.

We investigated the utility of ctDNA detection in hepatoblastoma using a customized approach. About half of hepatoblastomas exhibit large deletions within the *CTNNB1* exon 3 locus (up to about 1000 bp) [9,10,11], and the detection of ctDNA SVs, including deletions, necessitates innovative strategies given the fragmented nature of ctDNA. To overcome this challenge, our assay was based on single-primer extension capture in addition to UMI incorporation, and a customized in-house SV calling method and software were developed to detect deletions in *CTNNB1* from short DNA sequences to improve the detectability of *CTNNB1* deletions. In addition to error correction, UMIs allowed a more accurate determination of ctDNA levels from VAF [29,30]. Under this framework, QUENCH achieved an LoD of 0.1% VAF and an LoB of 0% VAF with clinically relevant DNA input amounts of 20 ng (equivalent to 0.5 mL plasma volumes, which is the typical volume of blood samples that children with hepatoblastoma can provide [25]).

One major advantage of our NGS-based method is its applicability to nearly all patients with hepatoblastoma, unlike patient-tailored ddPCR assays that require a priori knowledge of the somatic events from tumor tissues and time to design individual assays accordingly. Notably, two of the patients in our study had a provisional diagnosis based on imaging and AFP levels and commenced neoadjuvant chemotherapy to manage their symptoms without a histological conformation of hepatoblastoma. *CTNNB1* ctDNA variants were detected in both patients at this time point. However, an additional 2 and 8 months were needed to confirm these variants in the matched tumor samples using Sanger sequencing. Primary tumors were resected 2 and 3 months post neoadjuvant treatment, with adequate tumor material for mutation discovery from only one of them, as the other was completely necrotic. Lung metastasis was available 8 months post diagnosis and was used to confirm the second variant.

It is worth discussing that the only case with undetectable ctDNA at disease diagnosis assessed by QUENCH was also the only patient with very low-risk disease. This case, however, did not have the lowest AFP levels; rather, a high-risk tumor was presented with the lowest AFP values. The high sensitivity to detect ctDNA at disease diagnosis (nine out of ten patients, 90%), even for localized cases (seven out of eight patients, 88%), makes ctDNA liquid biopsy a potentially important tool to refine risk stratification between the very low- and low-risk groups, although this requires further validation.

We observed an excellent correlation between AFP levels and tumor volume at diagnosis; however, very large tumors did not show an association between tumor volume and ctDNA levels, implying that additional factors contribute to ctDNA levels in the blood. Interestingly, AFP also faces sensitivity and specificity limitations in diagnosing hepatocellular carcinoma and detecting postoperative recurrence [31]. An additional point of note is the correlation of ctDNA VAF with tumor size and AFP levels in hepatocellular carcinoma, as well as an association between ctDNA positivity and macrovascular invasion found by Ge Z and colleagues [32]. The data for vascular invasion are unfortunately unavailable in our cohort.

We found that ctDNA is a good surrogate marker of tumor burden and have confirmed the capacity of serial ctDNA sampling to monitor dynamic tumor response, similarly to AFP. The ability of QUENCH to accurately quantify ctDNA VAF was demonstrated by both the high correlation with ddPCR for VAF above 0.3% and by the correlation between VAF and macroscopic residual disease at different clinical time points. On the other hand, based on the study results, the concentration of cfDNA in the plasma is not a good surrogate marker of ctDNA positivity as it did not reliably predict ctDNA positivity, especially in concentrations lower than 100 ng/mL. Nevertheless, it is crucial to acknowledge that the rarity of cases restricted our sample size and might limit the results from this study.

Potential disadvantages of ctDNA over AFP testing include longer turnaround times, increased assay costs, and limited sensitivity at a low disease burden. In addition, the implementation of NGS assays into clinical routine use can be limited by the complexity of the associated protocols and data analysis. While QUENCH was able to detect variants in clinical samples down to 0.3%, it had limitations in samples taken at low disease burden. To enable a near real-time method for testing ctDNA, our study employed a tumor-agnostic approach for *CTNNB1* ctDNA detection in which the LoD was determined from the HepG2 variant without patient-specific variant optimizations. When applied to clinical samples with different variants, QUENCH fell short of reliably detecting ctDNA at VAF in the range of 0.1–2.2% compared with ddPCR. In addition, QUENCH was unable to detect ctDNA in 4/5 samples taken at relapse, and ddPCR in 1/3 of them. Thus, QUENCH may lack the sensitivity required for clinical disease monitoring at low disease burden, such as with MRD.

Two important factors underlying the LoD of all ctDNA profiling methods are the number of cfDNA molecules recovered and the number of mutations in a patient’s tumor that are interrogated [30]. QUENCH was designed to capture variants in exon 3 of the *CTNNB1* gene, as it is a hallmark of sporadic hepatoblastoma, with single mutation genotyping confining ctDNA detection limits to 0.1% VAF. However, multigene panels assaying many variants instead of a single variant increase the probability of finding detectable variants in plasma samples and provide improved sensitivity relative to a single marker [30]. For example, Chaudhuri A and colleagues tracked multiple ctDNA variants in patients with stage I-III non-small-cell lung cancer following definitive treatment and reported that a 94% detection rate with multiple markers dropped to 58% using the same platform with only a single marker [33]. In the last few years, it has been recognized that targeted methylated ctDNA analysis demonstrates superior sensitivity and specificity to detect a broad range of cancers, compared with the mutation-based ctDNA assay [34]. Recently, the DNA methylation landscape of hepatoblastoma was profiled to find DNA methylation clusters which tightly correlate with histological subtypes, and clinical behaviors [11]. In addition, about 600 differentially methylated regions were identified by comparing the hepatoblastoma and their matched non-tumoral liver tissues [35]. Future improvements could involve the incorporation of methylation analysis to enhance QUENCH ctDNA detection sensitivity.

Combining ctDNA and AFP as complementary markers presents an intriguing avenue for enhanced diagnostic and prognostic precision in cancer management. While AFP has been a traditional biomarker for hepatoblastoma [20,36], its limitations in sensitivity and specificity underscore the need for adjunctive markers like ctDNA. This amalgamation may offer a multifaceted approach, leveraging both genetic and protein-level information, providing a more nuanced understanding of tumor dynamics and offering refined risk stratification strategies for personalized patient care in hepatoblastoma and other cancers where AFP and ctDNA play pivotal roles as biomarkers.

## 5. Conclusions

Changes to AFP values and imaging studies are the current mainstay for treatment monitoring and relapse surveillance in hepatoblastoma. Overall, our study provides promising evidence for the utility of quantitative NGS and ddPCR ctDNA detection as a surrogate marker of tumor burden and treatment response in this disease. Further investigations are warranted to elucidate the interplay between ctDNA and AFP and to define the optimal clinical applications for ctDNA- and AFP-based MRD detection and risk stratification.

## Data Availability

The data presented in this study are available on request from the corresponding author.

## Supplementary Materials

The following supporting information can be downloaded at: https://www.mdpi.com/article/10.3390/cancers16010012/s1, Figure S1: Schematic illustration of VAF estimation; Figure S2: QUENCH limit of detection and linearity; Figure S3: Schematic view of the CTNNB1 exon–intron structure targeted by QUENCH and the mutations in the studied cohort and HepG2 cell line; Table S1: Clinicopathological data and mutational status of the study cohort; Table S2: ddPCR primers/probes set information.
