# Supplementary figures and images for: Quantitative ctDNA Detection in Hepatoblastoma: Implications for Precision Medicine
Source: Cancers (Basel). 2023 Dec 19;16(1):12. doi: 10.3390/cancers16010012 (PMC10778269; doi:10.3390/cancers16010012)

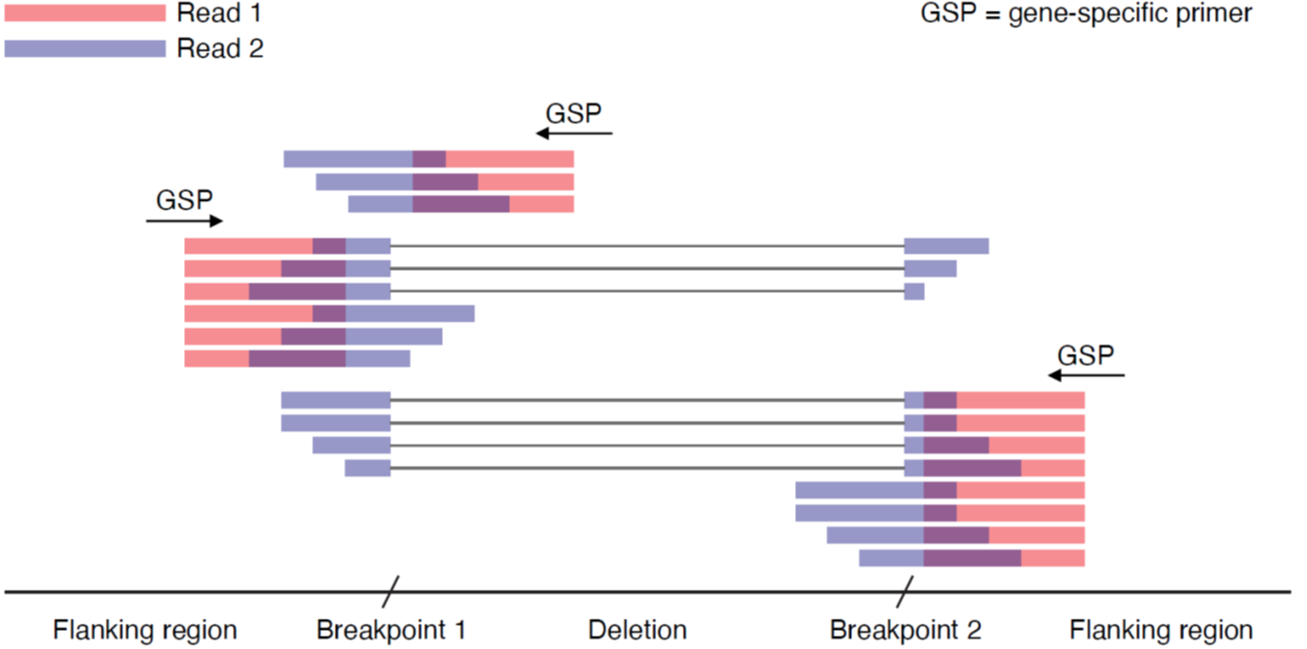

Supplement: Supplementary file 1 [file cancers-16-00012-s001.zip › Reviesed_Supplementary Materials/Figure S1a_new.tif]

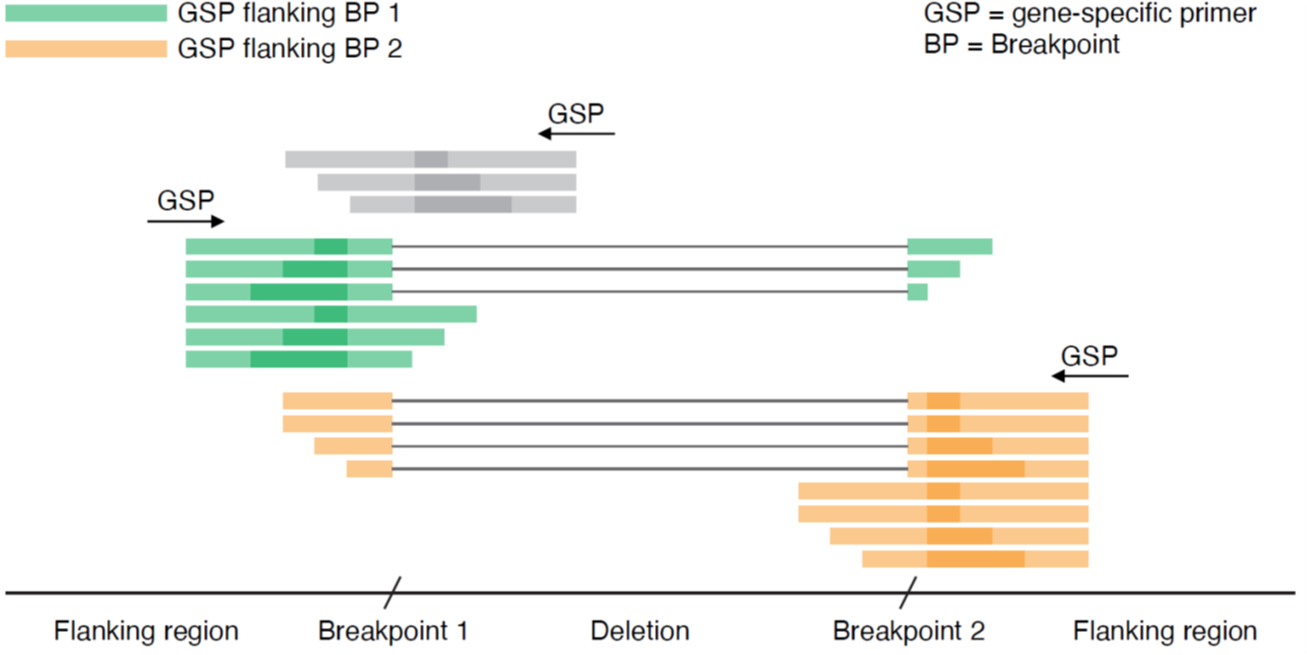

Supplement: Supplementary file 1 [file cancers-16-00012-s001.zip › Reviesed_Supplementary Materials/Figure S1b_new.tif]

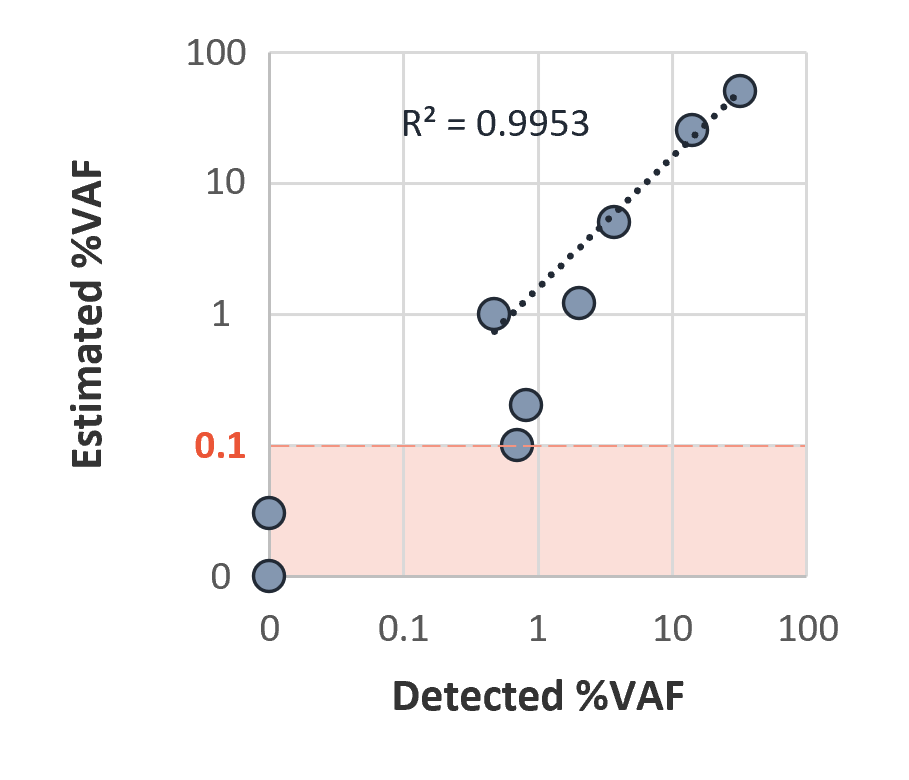

Supplement: Supplementary file 1 [file cancers-16-00012-s001.zip › Reviesed_Supplementary Materials/Figure S2_new.tif]

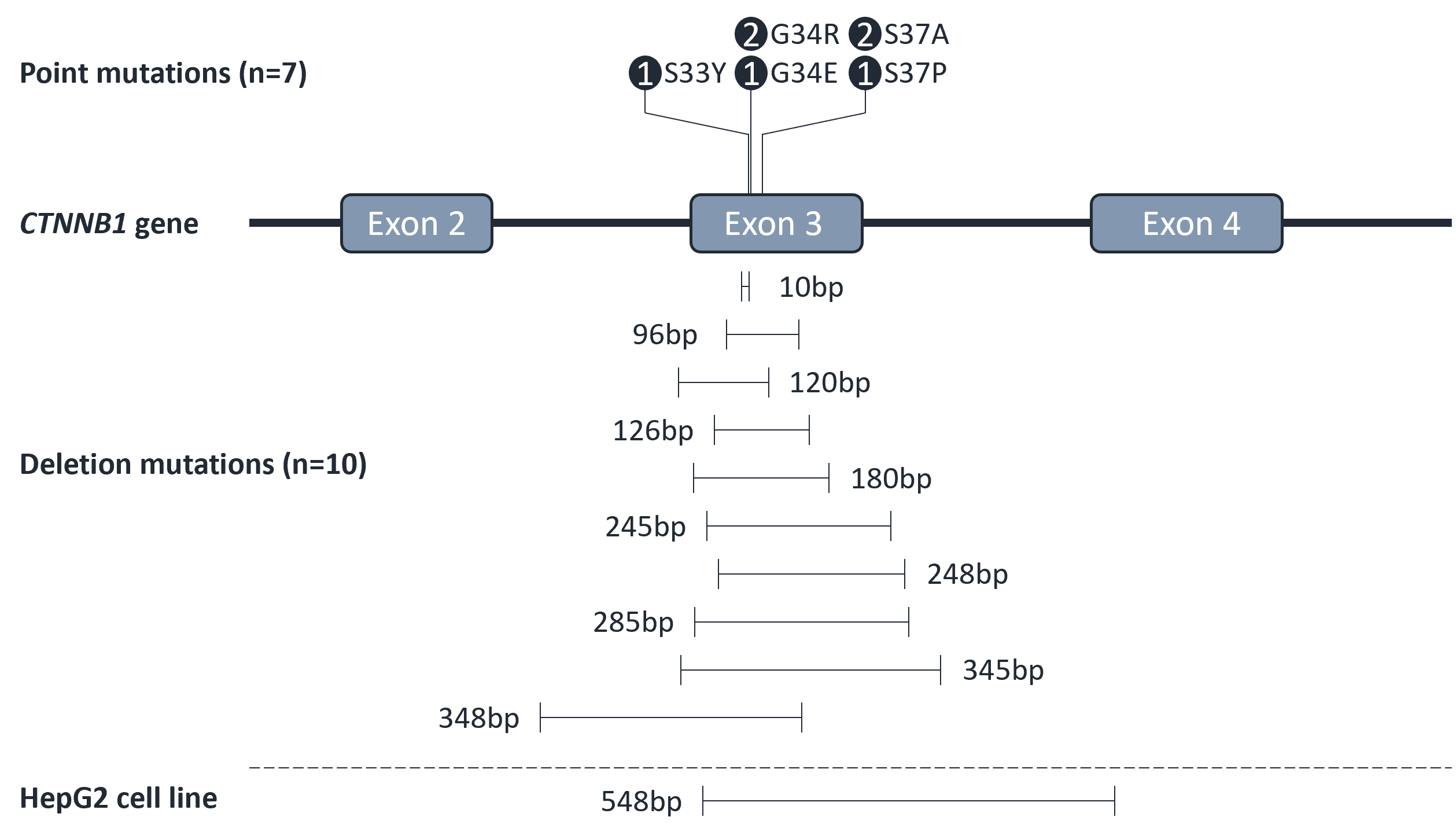

Supplement: Supplementary file 1 [file cancers-16-00012-s001.zip › Reviesed_Supplementary Materials/Figure S3_new.tif]
